# Super-multifactorial survey YHAB revealed high prevalence of sleep apnoea syndrome in unaware older adults and potential combinatorial factors for its initial screening

**DOI:** 10.3389/fragi.2022.965199

**Published:** 2022-10-14

**Authors:** Yuji Tanaka, Takashi Ando, Kazuki Mochizuki, Satoshi Igarashi, Kyoichiro Tsuchiya, Kozo Saito, Yasumi Ito, Zentaro Yamagata, Masaru Iwasaki, YHAB Health Data Survey Group 2020

**Affiliations:** ^1^ Department of Advanced Biomedical Research, Faculty of Medicine, University of Yamanashi, Yamanashi, Japan; ^2^ Yamanashi GLIA Centre, University of Yamanashi, Yamanashi, Japan; ^3^ Department of Orthopaedic Surgery, Faculty of Medicine, University of Yamanashi, Yamanashi, Japan; ^4^ Laboratory of Food and Nutritional Sciences, Department of Local Produce and Food Sciences, Faculty of Life and Environmental Sciences, University of Yamanashi, Yamanashi, Japan; ^5^ Department of Otorhinolaryngology, Faculty of Medicine, University of Yamanashi, Yamanashi, Japan; ^6^ Department of Diabetes and Endocrinology, Faculty of Medicine, University of Yamanashi, Yamanashi, Japan; ^7^ Department of Neuropharmacology, Faculty of Medicine, University of Yamanashi, Yamanashi, Japan; ^8^ Faculty of Engineering, University of Yamanashi, Yamanashi, Japan; ^9^ Department of Health Sciences, Faculty of Medicine, University of Yamanashi, Yamanashi, Japan; ^10^ Department of Clinical Research Collaboration Promotion, Faculty of Medicine, University of Yamanashi, Yamanashi, Japan

**Keywords:** sleep, daily steps, apnoea-hypopnoea index, older adults, healthy longevity, respiratory frail, aging, apnoea syndromes

## Abstract

**Study Objectives:** Aging is a risk factor for sleep apnoea syndrome (SAS), which is associated with lower quality of life and sudden mortality. However, SAS is often overlooked in older adults without suspicions. Therefore, this study aimed to evaluate SAS incidence and 48 other general factors in older adults.

**Methods:** This cross-sectional study included all non-caregiver-certified, healthy individuals (N = 32) who survived during the long-term cohort study and agreed to participate in apnoea-hypopnoea index (AHI) measurement (aged 83–95 years). AHI and 48 other general factors were evaluated, and simple linear regression analysis was used to identify potential AHI-related factors. Stepwise evaluation was further performed using multiple linear regression analyses.

**Results:** Although no individuals were previously diagnosed with SAS, 30 (93.75%) participants had some degree of SAS (AHI > 5/h), and 22 (68.75%) had severe or moderate SAS (AHI > 15/h). Compared with typical single risk factors represented by body mass index, combining daily steps and other factors improved the fit to the multiple linear regression. Combining daily steps and body mass index improved the fit for males and combining daily steps and red blood cell count improved the fit for females.

**Conclusion:** SAS was highly prevalent in unaware healthy Japanese older adults; combinations of daily steps and body mass index, and daily steps and red blood cell count may predict AHI in such individuals without the need for a specific AHI test.

## 1 Introduction

Sleep apnoea syndrome (SAS) is associated with decreased quality of life (Baldwin et al., 2001) (e.g., drowsiness that could cause traffic accidents (Findley et al., 1989) and sudden mortality (Yaggi et al., 2005) in all generations and with cognitive function in older adults (Ju et al., 2012). A major risk factor for SAS is aging (Young et al., 2002; Jordan et al., 2014; Matsumoto et al., 2020). Therefore, the number of patients with SAS is expected to increase as life expectancy increases worldwide. Continuous positive airway pressure (CPAP) is a typical treatment method for SAS (Loube et al., 1994; Aurora et al., 2012) and has been shown to reduce mortality (Martínez-García et al., 2012; Jennum et al., 2015; Dodds et al., 2020). However, it is difficult to suspect SAS, especially in those who sleep alone. There is a need for a simple screening method for SAS that can be widely used for the early detection and treatment of older adults.

Body mass index (BMI) is an important risk factor of SAS (Yaggi et al., 2005; Hudgel et al., 2012; Fan et al., 2019). However, it is difficult to assess SAS using only BMI or age because *R* ^2^ age and BMI are associated with various diseases and are not specific to SAS. Other risk factors include snoring (Martínez-García et al., 2012; Heinzer et al., 2015), high levels of high-density lipoprotein (HDL), cholesterol (Nadeem et al., 2014), high red blood cell count (Feliciano et al., 2017), smoking (Yaggi et al., 2005; Martínez-García et al., 2012), and male sex (Martínez-García et al., 2012) have also been reported as risk factors of SAS, but these factors cannot individually replace age or BMI (Young et al., 2002; Hudgel et al., 2012; Martínez-García et al., 2012). In addition, many of these factors are associated with obesity and metabolic complications; however, recent data (Matsumoto et al., 2020) showed that the presence of SAS might be unrelated to obesity in older adults aged 70–80 years. Thus, there may be unknown age-dependent SAS-related factors that are specific to older adults.

Therefore, creating and using a multifaceted survey data set for older adults is required to find suitable risk factors in this population, to establish efficient SAS screening. The multifaceted survey should incorporate recent technologies based on health data integration (Tang et al., 2006; Castillo et al., 2010; Widmer et al., 2013). However, many older adults are not dexterous with modern technologies such as smartphones and testing devices, and it is difficult for them to perform various measurements simultaneously, leading to incomplete coverage. In fact, only a few SAS studies have been reported in older adults aged >80 years (Young et al., 2002). Therefore, it is challenging to obtain multifaceted data regarding healthy longevity. In addition, recent mobile devices have made it possible to perform various calculations using many variables, which could enable SAS screening with adequate accuracy. When multiple risk factors are used for prediction, it is necessary to consider the optimal combination (Derksen and Keselman, 1992; Walter and Tiemeier, 2009), but there are few examples of such studies for SAS.

The apnoea-hypopnoea index (AHI) is a scale that measures the number of times breathing stops for 10 s or more per hour during sleep. AHI is the most important diagnostic indicator of SAS and is used to categorise the severity of the SAS. In this study, a multifaceted survey of healthy older adults was conducted to reveal the sets of general factors related to AHI for early detection of SAS in healthy older adults. The participants in this study included the survivors of a long-term cohort study conducted in the Yamanashi Prefecture (Kondo et al., 2008b; Kazama et al., 2011; Yamada et al., 2017). Japan has the highest life and healthy life expectancies worldwide. Interestingly, the Yamanashi Prefecture features the highest healthy life expectancy among all prefectures in Japan (Kapur et al., 2017; Naito et al., 2020).

This study aimed to obtain multifaceted data and identify the biological factors associated with high healthy life expectancies in the Yamanashi Prefecture (Yamanashi Healthy active long-living older people Biobank for healthy aging biosciences (YHAB). By analysing AHI-related factors in older adults, we also aimed to elucidate the factors that contribute to healthy longevity.

## 2 Methods

### 2.1 Study design, setting, and participants

**FIGURE 1:**
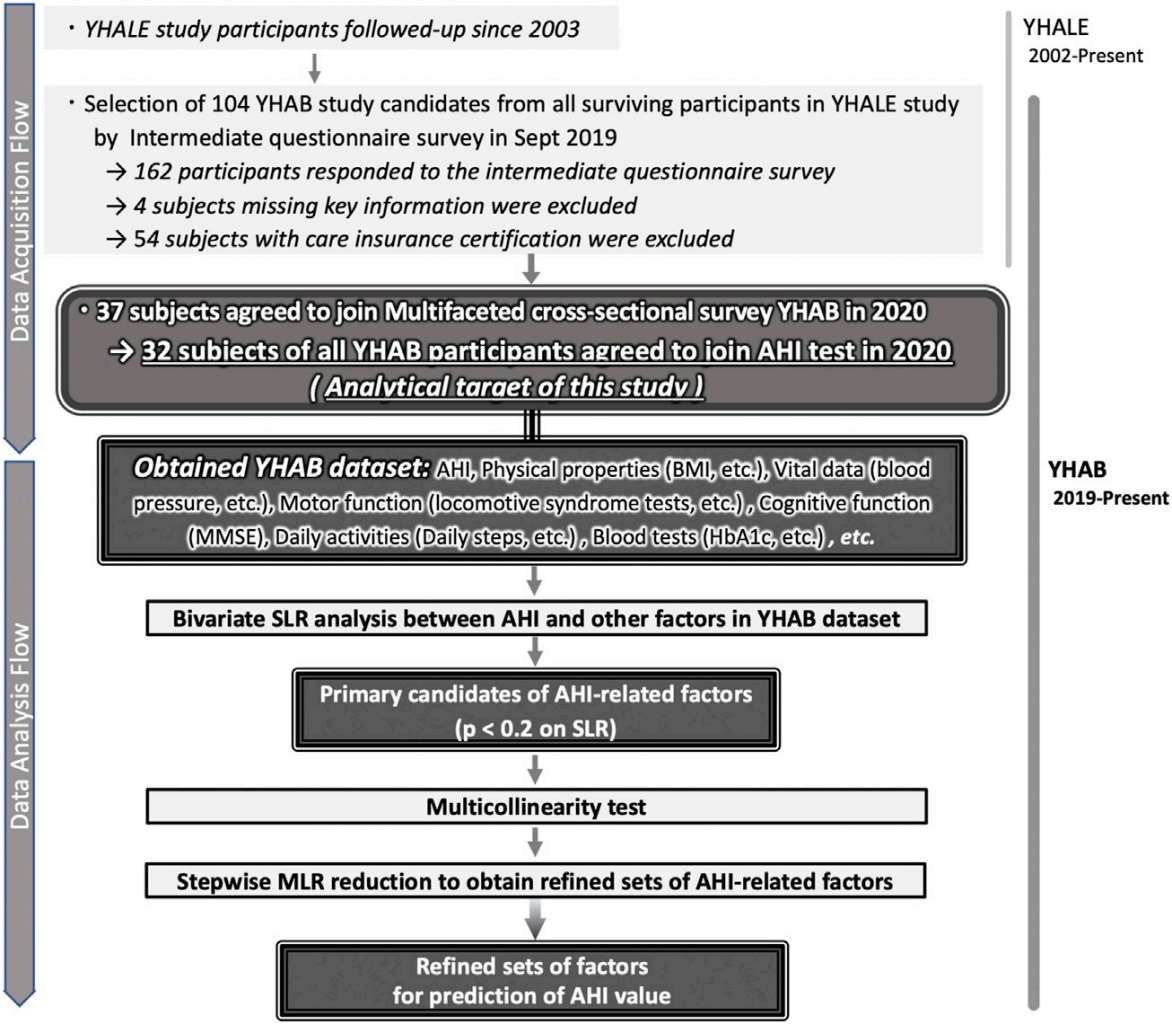
Selection of general factors in the study participants to estimate the AHI in older adults. (Top) Detailed description of the multifaceted, cross-sectional YHAB survey responses by the participants selected from among the survivors of the longitudinal Y-HALE study. (Bottom) Selection and refinements of the general factors to estimate the AHI from the YHAB datasets. AHI, apnoea-hypopnoea index; YHAB, Yamanashi Healthy Active Longevities Biobank for healthy aging biosciences; Y-HALE, Yamanashi Healthy-Active Life Expectancy; BMI, body mass index; SLR, simple linear regression; MLR, multiple linear regression.

The Y-HALE cohort study was initiated in 2003 with 587 participants (Figure 1). Since its initiation, the long-term care requirement, and assessments of activities of daily living over time have been monitored (Kondo et al., 2008b; Kazama et al., 2011). Of the 184 surviving participants in 2019, healthy older adults without certification for long-term care requirements were invited to participate in this 2020 YHAB study comprising a multi-item survey and various physical measurements. Of all the surviving participants, those who were able to respond to the intermediate questionnaire health survey with key information were included. Participants who were considered as needing long-term care under Japan’s long-term care insurance system were excluded from this study. In addition, for each individual survey item, if it was determined that there was a risk of a health problem with its implementation or if the subject did not wish to participate, he/she was excluded from the relevant test item. For example, if the subject had pain in the feet, he/she was excluded from the foot-based motor skills test.

All measurements including AHI were performed using a sleep apnoea testing device that further enabled SAS diagnosis. They were conducted cross-sectionally between January and December 2020, with an individual measurement schedule for each participant; all items were administered within 2 months. A two-step data analysis approach (extraction using the lax criteria and stepwise reduction) was used to identify the potential diagnostic predictors of SAS in healthy older adults.

### 2.2 Ethical considerations

This study was approved by the Ethics Committee of the University of Yamanashi School of Medicine (approval number: 2096 and date of approval: December 2019). After explaining the content of the study in writing and orally, those who gave their consent in writing were asked to participate in this study.

### 2.3 Measurements

#### 2.3.1 Basic physical measurements

The participants’ weight, body fat, muscle, and water percentages were measured using a body composition meter (Tanita MC-780A-N; Tanita Corp., Tokyo, Japan) (Yamada et al., 2017). Accurate weight values were measured by removing as much clothing as possible and subtracting the estimated weight of the remaining clothing from the actual measurement (assuming 1.0 kg for January and February and 0.5 kg for March through December). Height was measured using a stadiometer (Height Measurement HM 200P; Charder Electronic Co. Ltd., Taichung City, Taiwan). Systolic blood pressure, diastolic blood pressure, and pulse were measured using a sphygmomanometer (Terumo ES-W300ZZ; Terumo Corp., Tokyo, Japan).

#### 2.3.2 Sleep apnoea test (apnoea-hypopnoea index measurement)

AHI was measured using a portable monitoring device (WatchPAT 200; Itamar Medical, Caesarea, Israel) that records peripheral arterial tonometry signals, heart rate, oxygen saturation, and actigraphy (Naito et al., 2020). WatchPAT calculates clinical parameters, such as the respiratory event index and 4% oxygen desaturation index using an automated and proprietary algorithm. It is less burdensome for patients than full polysomnography and is recommended by the American Academy of Sleep Medicine guidelines for obstructive SAS (Kapur et al., 2017). The resulting data were automatically analysed to estimate respiratory events, such as AHI and respiratory disturbance index, and sleep states. This analysis has been described in greater detail elsewhere (Kinoshita et al., 2018).

#### 2.3.3 Daily step count

Daily steps were counted using a three-axis solid-state accelerometer (ActiGraph wGT3X-BT; ActiGraph Corp., Pensacola, FL, United States) (Ballin et al., 2020). The participants wore the device on the opposite wrist as that of the listener for 7 days during wakefulness and sleep, except during bathing or when the device felt uncomfortable.

#### 2.3.4 Grip strength measurement

Two measurements were taken for grip strength using a grip strength measuring device comprising a digital force gauge (product no. ZP-500N; IMADA, Toyohashi, Japan) and a computer/display system (Matsui et al., 2014). The mean peak grip strength was used for analysis.

#### 2.3.5 Locomotive syndrome risk test

The locomotive syndrome risk test (locomotive test) was used to detect locomotive syndrome (Hanyu et al., 2011; Yamada et al., 2020). This test comprises three parts as follows: the stand-up test, which evaluates the muscle strength required for standing up from seats of different heights; the two-step test, which evaluates the length of two strides; and the questionnaire with 25 questions regarding physical movement correlated with the European QOL Scale-5 Dimensions (EQ-5D) (Seichi et al., 2012). The risk level of the locomotive syndrome (locomotive score) was determined as previously described (Matsui et al., 2014; Seichi et al., 2012).

#### 2.3.6 Blood tests

Blood samples were collected using appropriate blood collection tubes (e.g., for plasma, clot formation, and whole blood). The samples were stored separately after centrifugation and other processing. The following laboratory values were evaluated by SRL, Tokyo, Japan, or Japan Medical, Yamanashi, Japan: blood urea nitrogen (BUN), creatinine, total cholesterol, HDL cholesterol, haemoglobin A1c (HbA1c), white blood cell count, red blood cell count, haematocrit, and cystatin C.

#### 2.3.7 Mini-mental state examination

The participants’ cognitive function was assessed by physicians using the Japanese version of the mini-mental state examination (MMSE) (Perneczky et al., 2006).

#### 2.3.8 Occlusal force measurement

The occlusal force was measured using a bite force measurement system (Dentalplescale, GC, Tokyo, Japan).

#### 2.3.9 Questionnaire on lifestyle

A questionnaire regarding lifestyle habits and medical history was completed by each participant, and the participant’s alcohol consumption rate, smoking rate, and history of SAS were evaluated.

### 2.4 Statistical analyses

Statistical analyses were performed as described in Figure 1 bottom using JMP® Pro 15.1.0 (SAS Institute Inc., Cary, NC, United States). First, we conducted a bivariate relationship analysis of the association of all parameters with continuous AHI values using simple linear regression (SLR). Second, selected AHI-related factors (with and without blood factors) were reduced by stepwise evaluation using multiple linear regression (MLR) (Derksen and Keselman, 1992; Walter and Tiemeier, 2009). Locomotive score was used as a dummy number. Participants with missing data were excluded for each analysis and the remaining data was used (n, number of data points used). *R* ^2^ was calculated to evaluate data fitting to the SLR. The estimation value (β) and *p*-value were calculated to estimate each explanatory variable.

The International Classification of Sleep Disorders (ICSD) was used to categorize SAS severity.

## 3 Results

### 3.1 Participants and data collection

Overall, 37 of the 104 healthy older people with no certification of care requirement (35.6%) agreed to participate in the multifaceted YHAB survey in 2020 (Figure 1). Of these, 32 participants (86.5%) underwent the sleep apnoea test. A total of 48 additional factors were evaluated, of which 46 were obtained from all 32 participants (Table 1; Supplementary Table S1). The remaining two factors (locomotive stand-up and two-step test) were evaluated in 30 participants; two participants who had physical problems were excluded.

**TABLE 1:** Representative dataset obtained by supra-multidimensional surveys of Japanese older adults residing in Yamanashi Prefecture (N = 32). The table shows a detailed breakdown of participants based on AHI severity.

Parameter	Mean	95% CI	SD	First IQ	Median	Third IQ	n
AHI (events/h)	23.2	17.7–28.8	15.4	12.1	20.7	32.5	32
Age (years)	86.7	85.5–87.8	3.2	84.0	86.0	87.8	32
BMI (kg/m^2^)	23.5	22.3–24.6	3.1	20.8	23.8	25.3	32
Fat mass (kg)	16.1	13.7–18.5	6.6	11.7	16.1	18.8	32
SBP (mmHg)	148.0	141.1–154.9	19.1	135.3	147.0	159.5	32
DBP (mmHg)	82.8	79.5–86.1	9.2	78.0	82.0	87.0	32
Locomotive two-step test value (two-step-distance/height)	1.1	1.0–1.2	0.3	1.0	1.2	1.3	30
Locomotive questionnaire (total score: 0–25)	8.2	3.6–12.8	12.7	1.0	3.0	9.5	32
Mean grip strength (kg)	25.3	21.5–29.1	10.5	16.7	23.9	36.1	32
Daily steps (steps/day)	8,871.1	7,646.3–10,095.8	3,397.0	5673.0	9574.0	11237.0	32
Alcohol consumption (all participants) (g/week)	88.0	58.0–118.0	83.2	0.0	0.0	63.9	32
Alcohol consumption (drinkers only) (g/week)	117.3	85.3–149.3	75.8	15.2	70.5	100.3	14
Cigarette consumption (all participants) (number of cigarettes/day)	1.2	−0.3–2.6	4.0	0.0	0.0	0.0	32
Cigarette consumption (smokers only) (number of cigarettes/day)	12.3	−4.6–29.2	6.8	7	10	20	3
Urea nitrogen (BUN) (mg/dL)	20.1	18.0–22.2	5.8	16.0	18.5	25.0	32
Total cholesterol (mg/dL)	205.2	193.4–217.1	32.9	180.3	208.5	220.8	32
HDL cholesterol (mg/dL)	62.5	57.0–68.0	15.3	51.0	60.0	73.8	32
HbA1c (NGSP)	6.1%	5.8%–6.4%	0.8%	5.6%	5.9%	6.4%	32
White blood cell count (cells /μL)	5,650.0	5,179.5–6,120.5	1,305.1	4425.0	5550.0	6575.0	32
Red blood cell count (10^4^ cells/μL)	413.8	396.4–431.3	48.3	380.8	415.5	441.3	32
Haemoglobin (g/dL)	13.2	12.6–13.7	1.5	12.6	13.1	14.5	32
Haematocrit (%)	39.1%	37.6%–40.6%	4.1%	35.4%	39.7%	42.6%	32
Cystatin C (mg/L)	1.2	1.1–1.4	0.3	1.0	1.2	1.3	32

SD, standard deviation; CI, confidence interval; IQ, interquartile; AHI, apnoea-hypopnoea index; BMI, body mass index; SBP, systolic blood pressure; DBP, diastolic blood pressure; HDL, high-density lipoprotein; SD, standard deviation; BUN, blood urea nitrogen; CI, confidence interval; SAS, sleep apnoea syndrome; HbA1c, haemoglobin A1c. The number of participants in each group, mean value, 95% CI, and SD of each measurement are presented.

**FIGURE 2:**
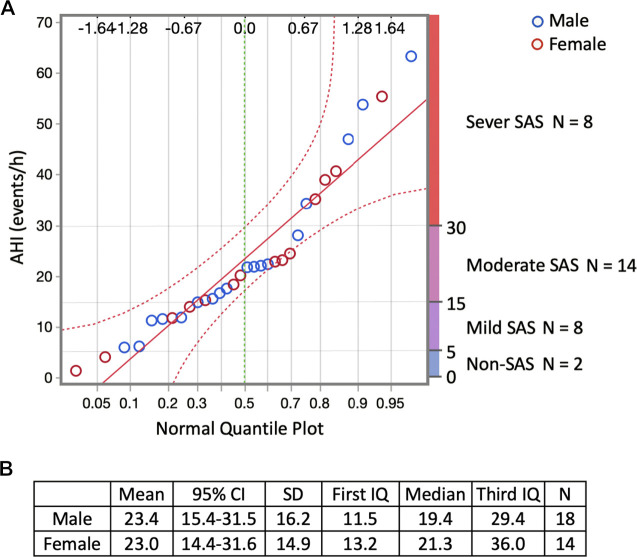
Responses regarding sleep-disordered breathing by the participants who responded to the YHAB survey. **(A)** Normal quantile plot of AHI and sleep-disordered breathing categories established by the ICSD. Blue circles indicate males, red circles indicate females. The upper abscissa indicates the normal quantile scale. Lower abscissa shows the cumulative empirical probability of each value. **(B)** Sex-specific dataset of the AHI. YHAB, Yamanashi Healthy Active Longevities Biobank for healthy aging biosciences; AHI, apnoea-hypopnoea index; ICSD, International Classification of Sleep Disorders; SAS, sleep apnoea syndrome; CI, confidence interval; SD, standard deviation.

The mean AHI of all participants was 23.2/h (Table 1; Supplementary Table S1). Regarding sleep-disordered breathing categories established by the ICSD, 25%, 43.75%, 25%, and 6.25% of participants had a severe (AHI ≥ 30/h), moderate (30/h > AHI ≥ 15/h), mild, (15/h > AHI ≥ 5/h) and non-SAS (5/h > AHI) condition, respectively (Figure 2A). Of all the participants, 68.75% had undiagnosed severe or moderate SAS (AHI > 15/h). The difference in the mean AHI between men (23.4/h) and women (23.0/h) was 0.4/h, and the difference was not significant (Figure 2B). No participant was diagnosed with SAS or received CPAP treatment prior to the study.

### 3.2 Screening of apnoea-hypopnoea index-related factors using bivariate relationship analysis with simple linear regression

The results of the bivariate relationship analysis with SLR between continuous AHI values and all 48 factors (Figure 1) are presented in Supplementary Table S2. Furthermore, the screened AHI-related factors with lax criteria (*p* < 0.2) are summarised in Table 2. Daily steps (*p* = 0.009) and the locomotive questionnaire (*p* = 0.034) showed the first and second most significant relationship with the AHI (*p* < 0.05), respectively. Daily steps, cystatin C, BUN, grip strength, and locomotive two-step values were negatively correlated with the AHI. The locomotive questionnaire, haematocrit, red blood cell count, BMI, fat mass, haemoglobin, and weight were positively correlated with the AHI using SLR (*p* < 0.20) (Table 2). Similar trends were observed in the correlation analysis (Supplementary Figure S1).

**TABLE 2:** Apnoea-hypopnoea index (AHI)-related factor pool of continuous AHI values.

Explanatory variables for AHI	n	R^2^	Estimation value	Standardised β	*p*-value
Daily steps	32	0.206	−0.002	−0.454	0.009
Locomotive questionnaire (total score)	32	0.141	0.455	0.375	0.034
Haematocrit (%)	32	0.114	1.260	0.338	0.058
Cystatin C	32	0.110	−16.534	−0.332	0.064
Red blood cell count	32	0.109	0.106	0.331	0.065
BMI	32	0.105	1.612	0.324	0.070
Fat mass	32	0.092	0.703	0.303	0.092
Blood urea nitrogen (BUN)	32	0.088	−0.785	−0.297	0.099
Haemoglobin	32	0.083	2.888	0.289	0.109
Weight	32	0.080	0.474	0.283	0.117
Mean grip strength	32	0.064	−0.372	−0.253	0.162
Locomotive two-step test value	30	0.067	−14.954	−0.259	0.167

Index values were selected from 48 parameters by simple linear regression using the lax criteria (*p* < 0.2). SBP was excluded because most participants on antihypertensive medication could not be excluded. BMI, body mass index; SBP, systolic blood pressure; DBP, diastolic blood pressure; HDL, high-density lipoprotein.

### 3.3 Stepwise refinement of apnoea-hypopnoea index-related factors

**FIGURE 3:**
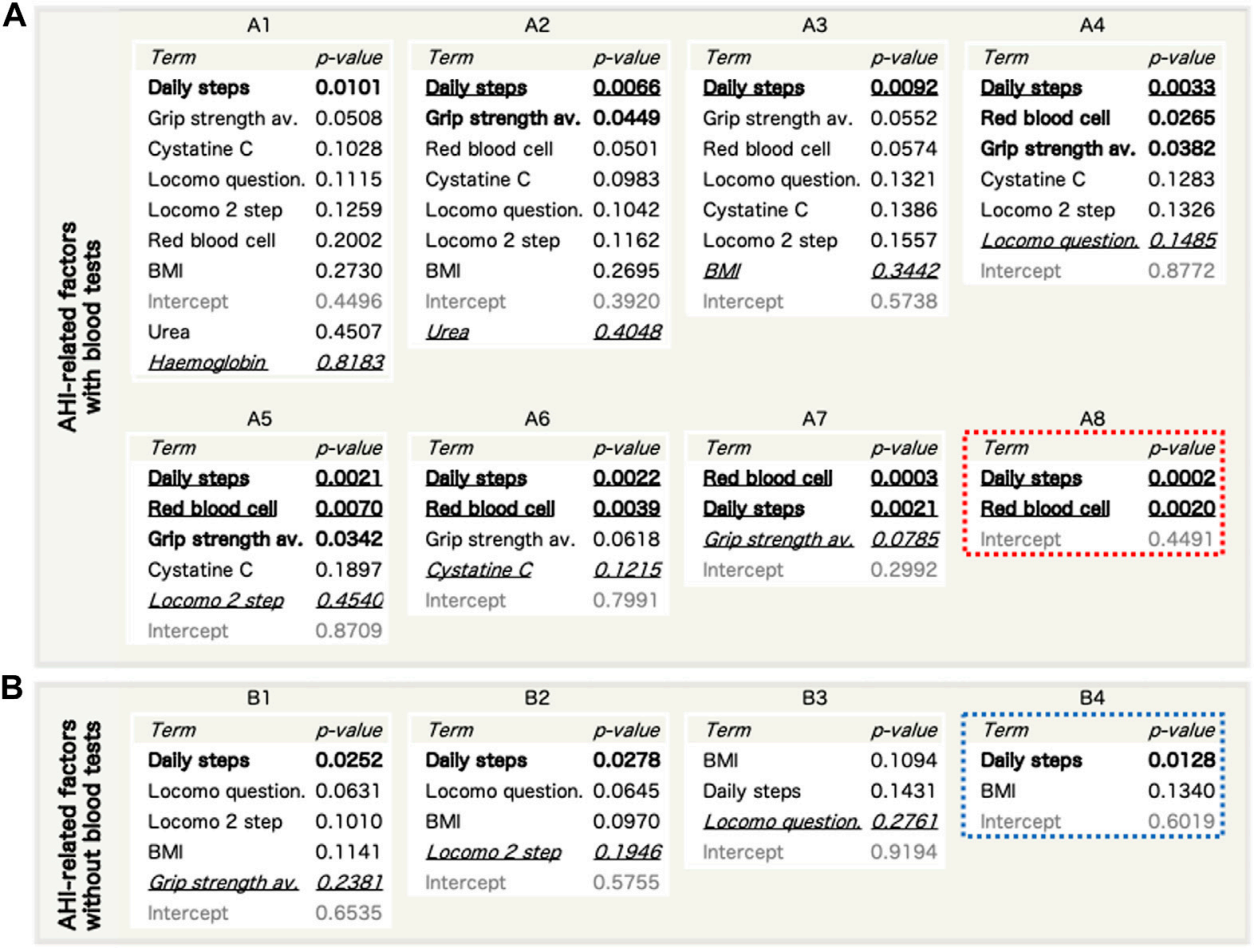
Refinement of AHI-related factors by stepwise reduction of used factors using a multiple linear regression (MLR) model. **(A,B)** The entire steps of reduction for AHI-related factor sets with blood factors (a, sets A1–8) and without blood factors (b, sets B1–4). Bold: *p* < 0.05. Bold and underlined: *p* < 0.01. Italic and underlined: the worst *p*-values in each set that were excluded in the next step. c, Refinement of AHI-related factor sets without blood factors. AHI, apnoea-hypopnoea index; BMI, body mass index.

The primary candidates (Table 2) were evaluated using MLR (Supplementary Table S3) to confirm multicollinearity. The haematocrit related to the red blood cell count and the fat mass related to the BMI were excluded to achieve a variance information factor of <10 (Supplementary Table S4). Next, we performed a stepwise refinement with MLR and removed the factors with the highest *p*-value on set A1 (Supplementary Table S5) to obtain sets A2–8 (Figure 3A). Additionally, we performed a stepwise refinement on set B (Supplementary Table S6), excluding blood-derived factors in set A1, to identify the refined factors in sets B2−4 without measuring blood parameters (Figure 3B). Daily steps and red blood cell counts were significantly associated with the AHI in the final model (A8). Regarding set B, daily steps and BMI were selected in the final model (B4).

### 3.4 Further evaluation of the final refined pairs of apnoea-hypopnoea index-related factors using multiple linear regression

**FIGURE 4:**
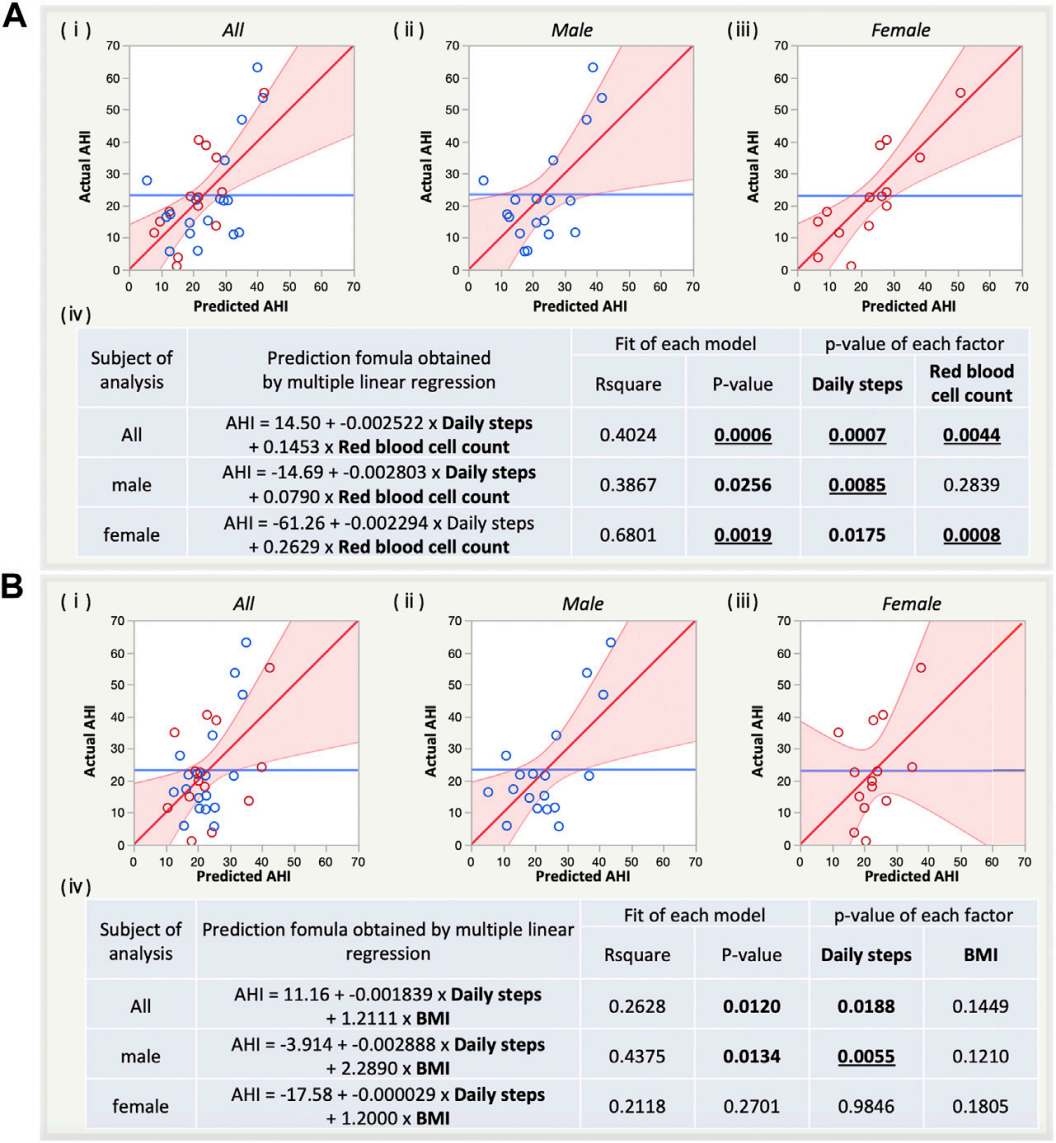
Sex-specific estimation of AHI by multiple linear regression (MLR) with the refined pairs of AHI-related factors with [**(A)**, a, set A8] and without blood factors [**(B)**, a, set B4]. (i) Prediction plot for both sexes, males, and females. (ii) Summary table for sex-specific estimation of AHI by MLR with the refined pairs. AHI, apnoea-hypopnoea index; BMI, body mass index.

Both AHI predictions using set A8 (Figure 4A) and set B4 (Figure 4B) tended to have lower *p*-values for the overall model for men and women combined than for sex-specific prediction. In contrast, the *R* ^*2*^ for the overall model was larger for women than for men in set A8 and was larger for men than for women in set B4. Regarding prediction for women in set A8, red blood cell count showed lower *p*-values compared with daily steps. Regarding prediction for men in set B4, daily steps showed lower *p*-values compared with BMI.

## 4 Discussion

In this study, a multimodal YHAB survey was administered to healthy older adults who had not been previously diagnosed with SAS. The results revealed that 68.75% of these participants had undiagnosed severe or moderate SAS (AHI>15/h). This allowed us to screen AHI-related factors and refine them to final pairs (sets A8 and B4). The final pairs improved the fit of the linear regression model for AHI estimation compared with the typical risk factor BMI. To the best of our knowledge, this study is the first to report that daily steps is a risk factor for SAS, as it played the most pivotal role in all the considered sets.

Concerning the ICSD criteria, 93.75% of the participants in this study had some degree of SAS (AHI > 5/h) without being aware of it. This is a novel finding of our study, and our results were more significant than those of a study conducted in the United States (Young et al., 2002). Our findings indicate the difficulties in the awareness of SAS, which may lead to social problems, such as increased traffic accidents caused by older adults (Findley et al., 1989). Since testing and treatment such as CPAP are generally well established for SAS (Loube et al., 1994; Aurora et al., 2012; Martínez-García et al., 2012; Jennum et al., 2015; Dodds et al., 2020), it is clinically essential to develop screening methods for patients with suspected SAS to improve the current situation in which this large number of elderly people are unaware of and untreated for SAS. A simple observation-based checklist was used to screen SAS in older adults (Onen et al., 2008); however, it must be conducted by a third party, such as a nurse. Daily steps and BMI, obtained in the final model B4, are the most widely used indices worldwide. Red blood cell count, obtained in set A8, is also routinely assessed in hospitals and various places (Matsui et al., 2014; Yamada et al., 2020). These could be used as general initial screening for SAS that can be utilized by the older adults themselves and their families.

The YHAB dataset was extremely diverse compared with datasets collected in previous studies of SAS among older adults (Loube et al., 1994; Young et al., 2002; Yaggi et al., 2005; Aurora et al., 2012; Hudgel et al., 2012; Ju et al., 2012; Martínez-García et al., 2012; Jordan et al., 2014; Heinzer et al., 2015; Jennum et al., 2015; Fan et al., 2019; Dodds et al., 2020; Matsumoto et al., 2020). Such diverse data are essential to identify novel AHI-related factors. To the best of our knowledge, this is the first time that a decrease in daily steps was found to be the most important risk factor for SAS in both SLR screening and stepwise MLR reduction analysis. Interestingly, despite the small number of participants in our study, most other primary candidates had known risk factors related to obesity (Loube et al., 1994; Young et al., 2002; Yaggi et al., 2005; Aurora et al., 2012; Hudgel et al., 2012; Ju et al., 2012; Martínez-García et al., 2012; Jordan et al., 2014; Heinzer et al., 2015; Jennum et al., 2015; Fan et al., 2019; Dodds et al., 2020; Matsumoto et al., 2020) or hypoxia (Fan et al., 2019). These facts strongly support the validity of the statistical approach used in our study and the results we obtained, including the finding of daily steps.

In this study, the number of daily steps was the most pivotal factor and showed a synergistic effect in combination with other factors related to obesity and hypoxia to distinguish older adults with SAS. Previous studies have shown that the positive effect of exercise on SAS improvement is independent of BMI (Peppard and Young, 2004; Sengul et al., 2011). In fact, obesity-related factors showed a weaker correlation with daily steps, and hypoxia-related factors showed less correlation with daily steps or BMI. These findings indicate that obesity, hypoxia, and physical activity are independently associated with SAS and complement each other to improve the accuracy of SAS detection.

Recent data (Matsumoto et al., 2020) clearly showed the presence of BMI-independent SAS in older adults aged 70–80 years; however, this has not been noted, explained, or discussed. Physical inactivates, a BMI-independent SAS-related factor identified in our study, may be key to elucidating this. Older adults with fewer steps were more likely to have decreased motor functions, such as grip strength and stride length, which are involved in a condition known as locomotive syndrome or frailty. Thus, it is reasonable to suggest that the excessively high prevalence of SAS in older adults can be attributed to SAS due to frail-like motor function in the respiratory system, in addition to obesity. Therefore, physical activity and motor functions, in addition to obesity, should be fully considered to distinguish older adults with SAS.

The age of the YHAB participants (86.7 years) was approximately 13 and 3 years more than the mean Japanese healthy life expectancy and life expectancy, respectively (Sato et al., 2019; GBD, 2020). However, the mean daily steps of the YHAB participants (8,871 steps) were nearly two-fold higher than those of the general Japanese older adults aged >65 years (5,396 and 4,656 steps for male and female participants, respectively) (Japanese Ministry of Health Law, Labour and Welfare, 2019), higher than those of the general Japanese adults aged >20 years (6,793 and 5,832 steps for male and female participants, respectively) (Japanese Ministry of Health Law, Labour and Welfare, 2019), and higher than those of the earlier older adults (mean age, 70.5 years) in Sweden (7,139 steps) (Yaggi et al., 2005). The locomotive tests also showed similar results for younger generations (Yamada et al., 2020). In addition, the 95% CIs for most factors were within the normal range. Notably, age was not associated with AHI in this group, despite age being reported as the main risk factor (Young et al., 2002; Jordan et al., 2014; Matsumoto et al., 2020), for which there is less need for statistical adjustment due to age. Exploratory studies with this unique dataset of healthy older adults will be beneficial to discover novel hypotheses for disease prevention and healthy longevity. As the most obvious example of this study, it is hypothesised that an increase in the daily steps of the general population will lead to a greater reduction in the AHI and a greater increase in healthy life expectancy. Such potential prospects need to be verified in large-scale studies, such as longitudinal and intervention studies.

This study has some limitations. First, the study population was limited to the YHAB participants, and the sample size was smaller than those of other SAS studies (Young et al., 2002; Jordan et al., 2014; Matsumoto et al., 2020). Nevertheless, to our knowledge, our data set contained the highest number of measurement items from older adults than other studies on average (Young et al., 2002; Jordan et al., 2014; Matsumoto et al., 2020). In addition to the highest number of items, the uniformity of the obtained data potentially compensates for the small sample size. However, additional validation is required to confirm the generalisability of our findings. Second, polysomnography was not performed in this study; polysomnography accurately calculates sleep time from the EEG data, whereas the WatchPAT used in this study calculates AHI based on the assumption that the measurement time is equivalent to the sleep time. Therefore, the AHI values obtained in our study are likely to be theoretically smaller than the true values. Further, we cannot rule out the possibility that the prevalence of SAS we identified may be higher. Third, although it has been reported that exercise improves SAS (Peppard and Young, 2004; Sengul et al., 2011), the causal relationship cannot be concluded in this study because it is possible that daytime sleepiness and other daily activities are reduced because of SAS. Fourth, the step counts using activity meters do not always accurately reflect the number of steps taken, because although the measuring device was worn on the dominant arm, arm movements during non-walking may also be added.

Overall, this multifaceted survey study revealed that more than half of the study participants had a high risk of undiagnosed severe or moderate SAS (AHI > 15/h). Sleep apnoea is an important condition, but all older adults should be screened for sleep difficulty, and the diagnosis should be considered in such older adults and should not just be based on the history of the patient and a bed partner. The findings of this study suggest that a combination of general indicators may predict AHI in these individuals without the need for a specific AHI test. These findings could promote SAS awareness among older adults.

## YHAB Health Data Survey Group 2020

Daisuke Ando, Naana Baba-Mori, Hirotaka Haro, Yusuke Iwata, Kenji Kashiwagi, Schuichi Koizumi, Atsuhito Nakao, Tomokazu Matsuoka, Masaki Omata, Katsue Suzuki-Inoue, Koichiro Ueki.

## Data Availability

The original contributions presented in the study are included in the article/Supplementary Material, further inquiries can be directed to the corresponding author.
