# Supplementary material for: Super-multifactorial survey YHAB revealed high prevalence of sleep apnoea syndrome in unaware older adults and potential combinatorial factors for its initial screening
Source: Front Aging. 2022 Oct 14;3:965199. doi: 10.3389/fragi.2022.965199 (PMC9614315; doi:10.3389/fragi.2022.965199)
Supplement: Supplementary file 1 [file Table5.pdf]

Supplementary Table 5. MLR analysis for set A1 that resulted from the exclusion of the primary candidates of AHI-related factors.

Explanatory variables for AHI	Estimate	Std Error	Wald Chi Square	Prob > Chi Square	Lower 95%	Upper 95%	VIF
**Daily steps**	**-2.354E-03**	**9.150E-04**	**6.619**	**0.010**	**-4.147E-03**	**-5.607E-04**	**1.916**
Mean grip strength	-0.521	0.267	3.813	0.051	-1.043	0.002	1.509
Cystatin C	-13.219	8.103	2.662	0.103	-29.100	2.661	1.287
Locomotive questionnaire (total score)	0.448	0.282	2.533	0.112	-0.104	1.000	2.659
Locomotive 2 step value (raw data)	23.004	15.031	2.342	0.126	-6.456	52.463	3.507
Red blood cell count	0.130	0.101	1.641	0.200	-0.069	0.329	4.470
BMI	0.850	0.776	1.202	0.273	-0.670	2.371	1.237
Intercept	-25.871	34.220	0.572	0.450	-92.941	41.198	0.000
Blood urea nitrogen (BUN)	0.349	0.462	0.569	0.451	-0.557	1.255	1.523
Hemoglobin	-0.745	3.241	0.0528	0.818	-7.098	5.609	4.719

BMI, body mass index; VIF, variance information factor; MLR, multiple linear regression; AHI, apnoea-hypopnoea index
