# Supplementary material for: Super-multifactorial survey YHAB revealed high prevalence of sleep apnoea syndrome in unaware older adults and potential combinatorial factors for its initial screening
Source: Front Aging. 2022 Oct 14;3:965199. doi: 10.3389/fragi.2022.965199 (PMC9614315; doi:10.3389/fragi.2022.965199)
Supplement: Supplementary file 2 [file Table4.pdf]

Supplementary Table 4. Exclusion of parameters from primary candidates to achieve a VIF of <10 to avoid multicollinearity.

	**Correlation of estimates**				VIF		Decision
Explanatory variables for AHI	***Hematocrit***	***Hemoglobin level***	***BMI***	***Fat mass***			
Hematocrit	**1.000**	**-0.827**	-0.054	0.047	***19.003***	→	Exclusion
Hemoglobin	**-0.827**	**1.000**	0.092	-0.106	***15.162***		
BMI	-0.054	0.092	**1.000**	**-0.952**	***13.156***		
Fat mass	0.047	-0.105	**-0.952**	**1.000**	***13.247***	→	Exclusion

BMI, body mass index; VIF, variance information factor.
