# Supplementary material for: Super-multifactorial survey YHAB revealed high prevalence of sleep apnoea syndrome in unaware older adults and potential combinatorial factors for its initial screening
Source: Front Aging. 2022 Oct 14;3:965199. doi: 10.3389/fragi.2022.965199 (PMC9614315; doi:10.3389/fragi.2022.965199)
Supplement: Supplementary file 3 [file Table1.pdf]

Supplementary Table 1. All dataset used for this study, which were obtained by supra-multidimensional surveys (YHAB) of Japanese older adults residing in Yamanashi Prefecture in 2020 (*N*=32).

Parameter	Mean	95% CI			SD	First IQ	Median	Third IQ	*n*
AHI (/h)	23.2	17.7	–	28.8	15.4	12.125	20.7	32.525	32
Age (years)	86.7	85.5	–	87.8	3.2	84	86	87.75	32
Height (cm)	154.3	151.7	–	156.9	7.2	148.75	155.15	159.675	32
Weight (kg)	56.0	52.7	–	59.3	9.2	48.2	55.85	63.675	32
BMI (kg/m^2)	23.5	22.3	–	24.6	3.1	20.825	23.75	25.275	32
Fat mass (kg)	16.1	13.7		18.5	6.6	11.675	16.1	18.775	32
Muscle mass (kg)	37.7	35.4		40.0	6.4	32.225	37.5	42.225	32
Water mass (kg)	29.1	27.4	–	30.8	4.8	25.75	28.8	31.375	32
SBP (mmHg)	148.0	141.1	–	154.9	19.1	135.25	147	159.5	32
DBP (mmHg)	82.8	79.5	–	86.1	9.2	78	82	87	32
Pulse (/min)	70.1	65.7	–	74.4	12.2	60	68.5	75	32
MMSE	26.2	25.1	–	27.4	3.2	24	27	29	32
Locomotive stand-up-test (categorised score: 0–3)	1.1	1.5	–	0.8	0.9	1	1	1.25	31
Locomotive 2-step-test value (2-step-distance/height)	1.1	1.0	–	1.2	0.3	1	1.2	1.325	30

Locomotive 2-step-test (categorised score: 0–3)	1.1	1.5	–	0.7	1.1	0	1	2	30
Locomotive questionnaire (total score: 0–25)	8.2	3.6	–	12.8	12.7	1	3	9.5	32
Locomotive questionnaire (categorised score: 0–3)	0.7	1.1	–	0.3	1.1	0	0	1	32
Mean grip strength (kg)	25.3	21.5	–	29.1	10.5	16.7125	23.925	36.0625	32
Mean occlusal force	34.6	29.8	–	39.4	13.1	31	36	40	31
Daily steps (/day)	8,871.1	7,646.3	–	10,095.8	3,397.0	5673	9574	11237	32
Alcohol intake (g/week)	88.0	58.0	–	118.0	83.2	0.0	0.0	63.9	32
Alcohol intake (drinkers) (g/week)	117.3	85.3	–	149.3	75.8	15.2	70.5	100.3	24
Cigarette consumption (/day)	1.2	-0.3	–	2.6	4.0	0.0	0.0	0.0	32
Cigarette consumption (smokers)(/day)	12.3	-4.6	–	29.2	6.8	7	10	20	3
Blood urea nitrogen (BUN) (mg/dL)	20.1	18.0	–	22.2	5.8	16	18.5	25	32
Uric acid (UA) (mg/dL)	5.7	5.3	–	6.1	1.2	4.85	5.85	6.475	32
Creatinine (mg/dL)	1.0	0.9	–	1.1	0.3	0.845	1.01	1.13	32
Total cholesterol (mg/dL)	205.2	193.4	–	217.1	32.9	180.25	208.5	220.75	32
HDL cholesterol (mg/dL)	62.5	57.0	–	68.0	15.3	51	60	73.75	32
Neutral fat (T-G) (mg/dL)	151.7	105.3	–	198.1	128.7	82.75	112.5	156.25	32

AST (GOT) (IU/L)	25.3	21.8	–	28.7	9.7	19.25	23	27.75	32
ALT (GPT) (IU/L)	19.3	17.0	–	21.6	6.4	14.25	18	23	32
γ-GT (γ-GTP) (IU/L)	27.0	19.4	–	34.6	21.1	15	21	28.75	32
Glucose (blood sugar) (mg/dL)	122.9	103.5	–	142.3	53.8	95.5	105	134.5	32
HbA1c (NGSP)	6.1%	5.8	–	6.4%	0.8%	5.6	5.9	6.425	32
White blood cell count (/μL)	5,650.0	5,179.5	–	6,120.5	1,305.1	4425	5550	6575	32
Red blood cell count (10^4/μL)	413.8	396.4	–	431.3	48.3	380.75	415.5	441.25	32
Hemoglobin (g/dL)	13.2	12.6	–	13.7	1.5	12.6	13.1	14.5	32
Hematocrit	39.1%	37.6	–	40.6%	4.1%	35.35	39.7	42.6	32
MCV (fL)	94.8	93.2	–	96.5	4.6	91.25	94.5	98	32
MCH (pg)	31.8	31.2	–	32.4	1.8	30.15	31.9	33.15	32
MCHC	33.6%	33.2	–	34.0%	1.1%	32.775	33.75	34.2	32
Platelet count (10^4/μL)	21.1	18.7	–	23.5	6.6	17	20.25	23.925	32
Neutrocytes	61.6%	58.5	–	64.6%	8.4%	54.375	62.8	69.575	32
Lymphocytes	28.7%	25.8	–	31.5%	8.0%	22.75	28.5	35.15	32
Monocytes	6.3%	5.7	–	6.9%	1.6%	5.3	6.35	7.5	32

Eosinophils	2.8%	2.1	–	3.5%	2.0%	1.225	2.45	3.575	32
Basophils	0.7%	0.5	–	0.8%	0.4%	0.4	0.6	0.9	32
Pepsinogen 1 (ng/mL)	68.4	53.0	–	83.7	42.5	40.1	60.85	92.475	32
Pepsinogen 2 (ng/mL)	14.3	11.5	–	17.2	7.9	8.65	12.05	17.55	32
Pepsinogen 1/2 ratio	4.8	4.0	–	5.7	2.3	3.225	4.75	5.775	32
Glycoalbumin	16.6%	15.3	–	17.9%	3.6%	14.125	15.75	18.4	32
Cystatin C (mg/L)	1.2	1.1	–	1.4	0.3	1.0275	1.16	1.335	32
Adiponectin (LA) (µg/mL)	14.9	12.6	–	17.1	6.2	11.375	14.15	17.25	32

SD, standard deviation; CI, confidence interval; IQ, Interquartile; AHI, apnoea-hypopnoea index; BMI, body mass index; SBP, systolic blood pressure; DBP, diastolic blood pressure; MMSE, mini-mental state examination; HDL, high-density lipoprotein; CI, confidence interval; AST, aspartate aminotransferase; GOT, glutamate oxaloacetate transaminase; ALT, alanine aminotransferase; MCV, mean corpuscular volume; MCH, mean corpuscular hemoglobin; MCHC, mean corpuscular hemoglobin concentration.
