# Supplementary material for: Super-multifactorial survey YHAB revealed high prevalence of sleep apnoea syndrome in unaware older adults and potential combinatorial factors for its initial screening
Source: Front Aging. 2022 Oct 14;3:965199. doi: 10.3389/fragi.2022.965199 (PMC9614315; doi:10.3389/fragi.2022.965199)
Supplement: Supplementary file 4 [file Table2.pdf]

Supplementary Table 2. Simple linear regression analysis to evaluate the relationship between all examined parameters and the contiguous apnoea–hypopnoea index (AHI) value.

Explanatory variables for AHI	n	R^2	Estimation Value	Standardised β	P-value
Age (years)	32	0.00073	0.1303	0.0271	0.883
Height (cm)	32	0.00472	0.1462	0.0687	0.709
Weight (kg)	32	0.07981	0.4744	0.2825	0.117
**BMI (kg/m^2)**	**32**	**0.10521**	**1.6116**	**0.3244**	**0.070**
**Fat mass (%)**	**32**	**0.09168**	**0.7030**	**0.3028**	**0.092**
Muscle mass (%)	32	0.00814	0.21709	0.09024	0.623
Water mass (%)	32	0.02641	0.51792	0.16251	0.374
**SBP (mmHg)**	**32**	**0.10114**	**-0.2561**	**-0.3180**	**0.076**
DBP (mmHg)	32	0.00805	-0.1508	-0.0897	0.625
Pulse (/min)	32	0.00652	0.1024	0.0807	0.661
MMSE	32	0.00686	0.4003	0.0828	0.652
Locomotive 2 step value (raw data)	30	0.06720	-14.9543	-0.2592	0.167
**Locomotive_questionnaire (total score)**	**32**	**0.14079**	**0.4545**	**0.3752**	**0.034**
Locomotive 2 step value (score)	30	0.05085	3.3639	0.2255	0.231

Locomotive questionnaire (score)	32	0.04643	3.0329	0.2155	0.236
Locomotive stand up (score)	30	0.03282	3.3354	0.1812	0.338
Mean grip strength	32	0.06419	-0.3716	-0.2534	0.162
Mean occlusal force	31	0.00187	0.0516	0.0432	0.817
**Daily steps (/day)**	**32**	**0.20575**	**-0.0021**	**-0.4536**	**0.009**
Alcohol intake a week (g/week)	32	0.02619	-0.0300	-0.1618	0.376
Cigarette consumption (/day)	32	0.46402	-1.1090	-0.6812	0.523
**Urea nitrogen (BUN) (mg/dL)**	**32**	**0.08798**	**-0.7847**	**-0.2966**	**0.099**
Uric acid (UA) (mg/dL)	32	0.00762	-1.1541	-0.0873	0.635
Creatinine (mg/dL)	32	0.04719	-11.3946	-0.2172	0.232
Total cholesterol (mg/dL)	32	0.03303	-0.0851	-0.1817	0.320
HDL cholesterol (mg/dL)	32	0.03929	0.1999	0.1982	0.277
Neutral fat (T-G) (mg/dL)	32	0.00974	-0.0118	-0.0987	0.591
AST (GOT) (IU/L)	32	0.01726	0.2089	0.1314	0.474
ALT (GPT) (IU/L)	32	0.03550	0.4517	0.1884	0.302
γ-GT (γ-GTP) (IU/L)	32	0.00224	0.0346	0.0473	0.797
Glucose (blood sugar) (mg/dL)	32	0.00794	0.0255	0.0891	0.628
HbA1c (NGSP)	32	0.01469	2.4330	0.1212	0.509
White blood cell count (/μL)	32	0.00740	0.0010	0.0860	0.509

**Red blood cell count (10^4/μL)**	**32**	**0.10928**	**0.1055**	**0.3306**	0.509
Hemoglobin (g/dL)	32	0.08324	2.8884	0.2885	0.509
**Hematocrit**	**32**	**0.11429**	**1.2599**	**0.3381**	0.509
MCV (fL)	32	0.00514	-0.2401	-0.0717	0.509
MCH (pg)	32	0.00441	-0.5779	-0.0664	0.509
MCHC	32	0.00034	-0.2577	-0.0186	0.509
Platelet count (10^4/μL)	32	0.00675	0.1915	0.0821	0.509
Neutrocytes	32	0.00015	0.0224	0.0122	0.509
Lymphocytes	32	0.00022	-0.0288	-0.0148	0.509
Monocytes	32	0.00224	0.4500	0.0473	0.509
Eosinophils	32	0.00017	-0.1021	-0.0131	0.509
Basophils	32	0.00970	-4.1412	-0.0985	0.509
Glycoalbumin	32	0.00051	-0.0962	-0.0227	0.509
**Cystatin C (mg/L)**	**32**	**0.11012**	**-16.5338**	**-0.3318**	0.509
Adiponectin (LA) (μg/mL)	32	0.00036	-0.0467	-0.0189	0.509

AHI, apnoea-hypopnoea index; BMI, body mass index; SBP, systolic blood pressure; DBP, diastolic blood pressure; MMSE, mini-mental state examination; HDL, high-density lipoprotein; CI, confidence interval; AST, aspartate aminotransferase; GOT, glutamate oxaloacetate transaminase; ALT, alanine aminotransferase; MCV, mean corpuscular volume; MCH, mean corpuscular hemoglobin; MCHC, mean corpuscular hemoglobin concentration.
