# Supplementary material for: Super-multifactorial survey YHAB revealed high prevalence of sleep apnoea syndrome in unaware older adults and potential combinatorial factors for its initial screening
Source: Front Aging. 2022 Oct 14;3:965199. doi: 10.3389/fragi.2022.965199 (PMC9614315; doi:10.3389/fragi.2022.965199)
Supplement: Supplementary file 5 [file Table6.pdf]

Supplementary Table 6. MLR analysis for set B1 comprising AHI-related factors excluding blood-derived factors from A1.

Explanatory variables for AHI	Estimate	Std Error	Wald Chi Square	Prob > Chi Square	Lower 95%	Upper 95%	VIF
**Daily steps**	**-0.002**	**0.001**	**5.013**	**0.025**	**-0.004**	**0.000**	**1.705**
Locomotive questionnaire (total score)	0.537	0.289	3.455	0.063	-0.029	1.102	2.332
Locomotive 2 step value (raw data)	25.548	15.575	2.690	0.101	-4.979	56.075	3.145
BMI	1.246	0.789	2.496	0.114	-0.300	2.792	1.068
Mean grip strength	-0.330	0.280	1.392	0.238	-0.878	0.218	1.385
Intercept	-11.317	25.210	0.202	0.653	-60.728	38.093	0.000

BMI, body mass index; VIF, variance information factor; MLR, multiple linear regression; AHI, apnoea-hypopnoea index
