# Supplementary material for: Super-multifactorial survey YHAB revealed high prevalence of sleep apnoea syndrome in unaware older adults and potential combinatorial factors for its initial screening
Source: Front Aging. 2022 Oct 14;3:965199. doi: 10.3389/fragi.2022.965199 (PMC9614315; doi:10.3389/fragi.2022.965199)
Supplement: Supplementary file 6 [file Table3.pdf]

Supplementary Table 3. Initial MLR analysis for primary candidates of AHI-related factors (P-value <0.2 on SLR, excluding SBP) to predict continuous AHI values.

Explanatory variables for AHI	Estimate	Std Error	Wald Chi Square	Prob > Chi Square	Lower 95%	Upper 95%	VIF
**Daily steps**	**-2.415E-03**	**9.303E-04**	**6.737**	**0.009**	**-4.238E-03**	**-5.912E-04**	**1.971**
Locomotive questionnaire (total score)	0.516	0.290	3.169	0.075	-0.052	1.083	2.798
Mean grip strength	-0.549	0.20	2.948	0.086	-1.175	0.078	2.157
Locomotive 2 step value (raw data)	25.594	15.326	2.789	0.095	-4.444	55.632	3.628
Cystatin C	-11.688	8.418	1.928	0.165	-28.188	4.811	1.383
Hematocrit	***2.827***	***2.389***	***1.400***	***0.237***	***-1.856***	***7.510***	***19.003***
Intercept	-65.213	55.668	1.372	0.241	-174.319	43.894	0.000
Hemoglobin	***-6.168***	***5.824***	***1.1212***	***0.290***	***-17.583***	***5.247***	***15.162***
BMI	***2.400***	***2.536***	***0.896***	***0.344***	***-2.570***	***7.371***	***13.156***
Blood urea nitrogen (BUN)	0.407	0.472	0.745	0.388	-0.518	1.333	1.580

Fat mass	***-0.7670***	***1.177***	***0.428***	***0.513***	***-3.076***	***1.537***	***13.247***
Red blood cell count	0.060	0.118	0.254	0.614	-0.172	0.291	6.025

MLR, multiple linear regression; SLR, simple linear regression; SBP, systolic blood pressure; AHI, apnoea-hypopnoea index.
