# Supplementary material for: Super-multifactorial survey YHAB revealed high prevalence of sleep apnoea syndrome in unaware older adults and potential combinatorial factors for its initial screening
Source: Front Aging. 2022 Oct 14;3:965199. doi: 10.3389/fragi.2022.965199 (PMC9614315; doi:10.3389/fragi.2022.965199)
Supplement: Supplementary file 7 [file Image1.pdf]

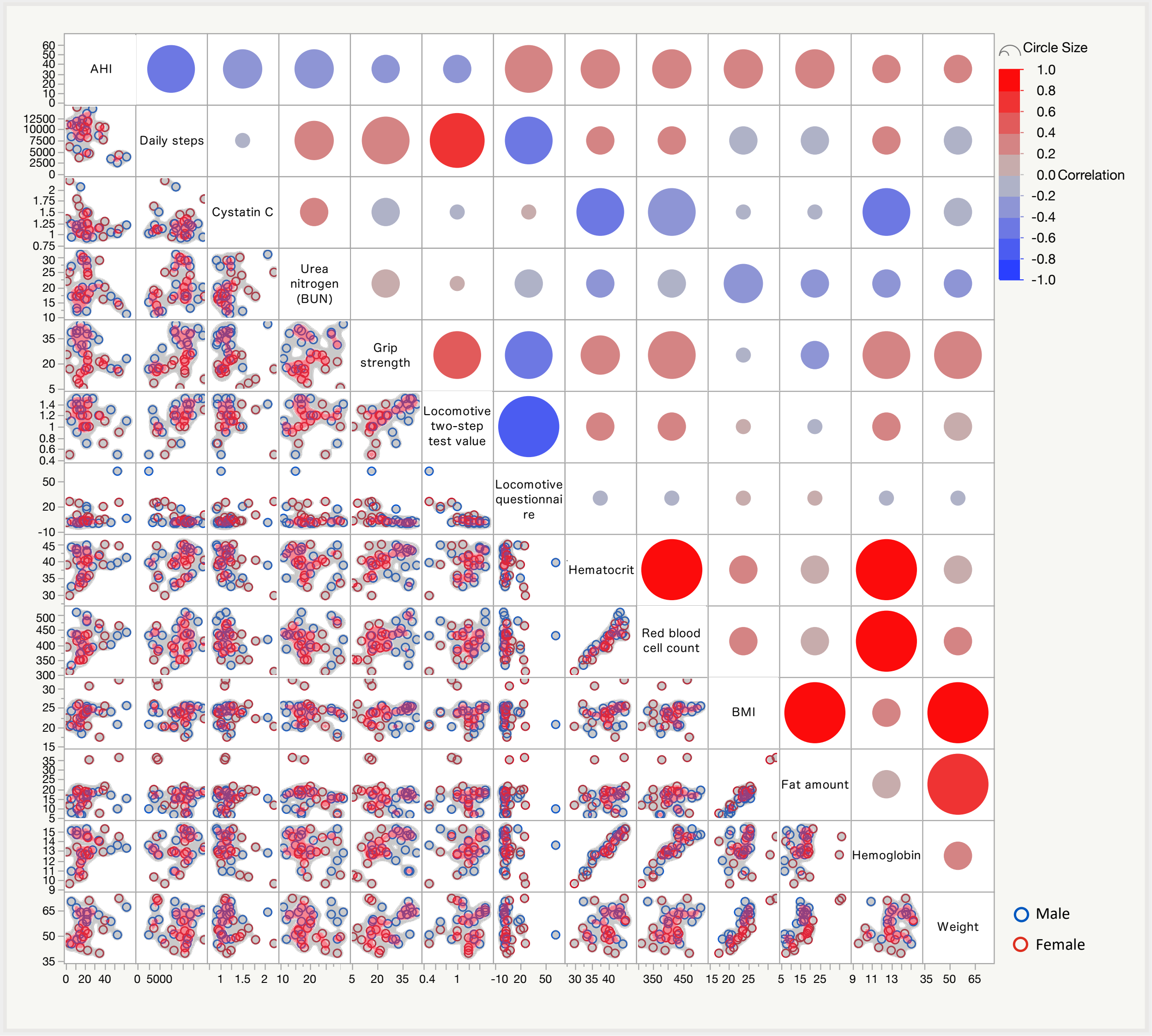

Supplementary Figure 1. Bivariate relation analysis to evaluate the correlation between the apnoea-hypopnoea index (AHI) continuous value and AHI-related factor pool (p-value < 0.2 on simple linear regression, excluding systolic blood pressure).
